# CXCR3 Expression Is Associated with Advanced Tumor Stage and Grade Influencing Survival after Surgery of Localised Renal Cell Carcinoma

**DOI:** 10.3390/cancers15041001

**Published:** 2023-02-04

**Authors:** Andrea Katharina Lindner, Agnieszka Martowicz, Gerold Untergasser, Johannes Haybaeck, Eva Compérat, Florian Kocher, Andreas Seeber, Martin Thurnher, Renate Pichler

**Affiliations:** 1 Department of Urology, Comprehensive Cancer Center Innsbruck (CCCI), Medical University Innsbruck, 6020 Innsbruck, Austria; 2 Tyrolpath Obrist Brunhuber GmbH, 6511 Zams, Austria; 3 Department of Internal Medicine V, Hematology and Oncology, Comprehensive Cancer Center Innsbruck (CCCI), Medical University Innsbruck, 6020 Innsbruck, Austria; 4 Institute of Pathology, Neuropathology and Molecular Pathology, Medical University Innsbruck, 6020 Innsbruck, Austria; 5 Diagnostic and Research Center for Molecular Biomedicine, Institute of Pathology, Medical University Graz, 8036 Graz, Austria; 6 Department of Pathology, Medical University of Vienna, 1090 Vienna, Austria; 7 Immunotherapy Unit, Department of Urology, Medical University Innsbruck, 6020 Innsbruck, Austria

**Keywords:** CXCR3 expression, chemokines, renal cell carcinoma, recurrence, immunohistochemistry, adjuvant immunotherapy, biomarker

## Abstract

**Simple Summary:**

Localized renal cell carcinoma is primarily treated surgically by resection. Some patients carry criteria for a high risk of tumour recurrence, for which postoperative immunotherapy is approved and currently used. The receptor CXCR3 differentiates anti-tumour T cells, which are known to be significantly increased in patients at high risk of tumor recurrence. The aim of our study therefore was to evaluate occurrence of CXCR3 in tissue samples, to analyse its expression in higher tumor grades and stages and to interpret the results to designate CXCR3 as a potential marker for predicting recurrence in renal cell carcinoma after primary surgical resection.

**Abstract:**

Background: Surgery is the standard treatment in localized renal cell carcinoma (RCC). Pembrolizumab is now approved for adjuvant therapy in high-risk patients. However, inhomogeneity of studies gives ambiguity which patient benefit most from adjuvant therapy. A high infiltration of CD8^+^ T cells is known to be linked with poor prognosis in RCC. CXCR3 is a key player of CD8^+^ T cell differentiation and infiltration. We aimed to evaluate CXCR3 as a potential marker for predicting recurrence. Methods: CXCR3 and immune cell subsets (CD4, CD8, CD68 and FoXP3) were measured on RCC samples by multiplex immunofluorescence (mIF) staining. Cellular localization of CXCR3 was evaluated using single-cell RNA analysis on a publicly available dataset. Results: Tumor samples of 42 RCC patients were analyzed, from which 59.5% were classified as clear-cell RCC and of which 20 had recurrence. Single-cell RNA analysis revealed that *CXCR3* was predominantly expressed in intratumoral T cells and dendritic cells. CXCR3 expression was higher in advanced tumors stages (*p* = 0.0044) and grade (*p* = 0.0518), correlating significantly with a higher CD8^+^ T cell expression (*p* < 0.001). Patients with CXCR3^high^ RCCs had also a significant shorter RFS compared to CXCR3^low^ (median: 78 vs. 147 months, *p* = 0.0213). In addition, also tumor stage pT3/4 (*p* < 0.0001) as well as grade G3/4 (*p* = 0.0008) negatively influenced RFS. Conclusion: CXCR3^high^ cell density was associated with high T cell infiltration and advanced tumor stage, worsening RFS in surgically resected RCC patients. Beside its prognostic value, CXCR3 might be a predictive biomarker to guide therapy decision for adjuvant therapy in localized RCC.

## 1. Introduction

Renal cell carcinoma (RCC) represents around 3% of all malignant visceral neoplasms with more than 400.00 newly diagnosed cases each year [1]. Clear-cell renal cell carcinoma (ccRCC) is the most frequent occurring histological subtype, representing around 75% of all RCCs [2,3]. Papillary RCC (pRCC) stands as the second most common subtype in 10% of cases [4,5], whereas chromophobe RCC represents about 5% of all malignant renal tumors. Surgical tumor resection remains the curative treatment of choice in localized RCC [1]. Nevertheless, 35–47% of patients will develop recurrence after primary surgery [6,7]. This group, decelerated as patients of high-risk of recurrence, has now more and more become the focus of new adjuvant therapy strategies.

The Keynote-564 trial led to a major paradigm shift for patients following surgical resection of localized RCC. It demonstrated the benefit of adjuvant pembrolizumab in intermediate or high-risk RCC [8], which is by now recommended by current guidelines [1,9]. Recently, publication of the 30-month follow-up (FU) data further supports the use of adjuvant pembrolizumab as a standard of care [10]. Nevertheless, data on overall survival (OS) are still pending and other trials failed to prove benefit of adjuvant immunotherapy (Checkmate 914 and IMmotion010 trial [11]). Moreover, no biomarkers are available to guide the clinical decision which patient will benefit most from adjuvant immunotherapy. 

The expression of CD8^+^ T cells is known to be significantly up-regulated in RCC [12], correlating significantly with poorer prognosis [13,14,15]. The identification of markers related to CD8^+^ T cell infiltration is needed to facilitate the monitoring of RCC immunotherapy response and the exploration mechanisms of immune cell infiltration. The C-X-C Motif Chemokine Receptor 3 (CXCR3) seems to be a key factor in influencing early programming of CD8^+^ T cell differentiation [16], yet not their migration [17]. Hickman et al. demonstrated that CXCR3-deficient CD8+ T cells presented with an impaired cytotoxicity and showed that CXCR3 plays a role in interaction between antigen-presenting cells and CD8+ T cells, allowing priming and further activation of T cells [18]. CXCR3 is preferentially expressed on activated CD8^+^ T cells and is also thought to be involved in increased trafficking of CD8^+^ T cells to tumors [19]. 

Chemokines cover a group of small functional secreted proteins, expressed in response to stimuli, such as TNFα or IFNγ [20]. They engage leucocyte subsets to local inflammatory sites. Among them, ligands of CXCR3, such as C-X-C motif ligand (CXCL) -9, -10 and -11 are known to attract different leucocyte subsets and to influence local inflammation and angiogenesis and proliferation [21]. Moreover they have potency in attracting anti-tumor T lymphocytes and may therefore mediate tumor growth inhibition and regression [22,23]. CXCR3 has two subtypes, CXCR3-A which induces chemotaxis and proliferation, favoring tumor growth and progression and CXCR3-B, inhibiting migration and inducing apoptosis, which may enhance anti-tumor immunity [24]. 

In RCC, published studies revealed an increased expression of CXCR3 and elevated concentrations of its ligands [25,26]. However, discrepancy between studies assessing the prognosis of patients in correlation of CXCR3 expression exists [27,28,29,30,31,32]. 

Herein, we analyzed CXCR3 expression on localized RCC tumor samples aiming to describe the immunological landscape as well as its prognostic value. 

## 2. Materials and Methods

### 2.1. Patient and Clinical Characteristics

Medical records of patients diagnosed with surgically treated primary localized non-metastatic RCC at our department were reviewed retrospectively from our RCC database. Patients with a confirmed histopathology of ccRCC or pRCC without previous history of oncological disease, eligible FU data at our outpatient department and viable histological samples were included. Patients with other simultaneous oncological diseases, those who were followed-up elsewhere postoperatively or who had evidence of distant metastasis at diagnosis were excluded. Consent of the local ethics commission of the Medical University Innsbruck was obtained with the approval number 1202/2018. According to the EAU guidelines [1], patients were treated with laparoscopic or open nephron-sparing partial nephrectomy whenever possible or laparoscopic nephrectomy when indispensable. The decision for defining the operative procedure was primarily based on the RENAL nephrotomy score, considered together with the patients age, renal function and allover co-morbidities. In elderly patients with impaired kidney function (>Grade 3a), the best possible kidney-preserving operation was attempted to prevent dialysis. Due to the difficult standardisation of the procedure decision, this was decided individually on base of the patient’s history and presentation to ensure an in toto tumor resection and to obtain the best possible remaining kidney function. All operations were performed by a small group of experienced surgeons in oncological renal surgery.

FU was performed including clinical visits and CT-scans on the basis of the Leibovich score predicting oncological outcome in RCC after surgery [33,34] as recommended and based on the EAU guidelines valid at the time of FU [1]. Disease progression was defined as local recurrence, or distant metastasis. 

### 2.2. Tumor Samples and Preparation

Formalin-fixed paraffin-embedded (FFPE) tumor samples of patients diagnosed with localized RCC at our institution were collected with regard to histological type, pathological stage and local recurrence. Tumor staging was performed according to the 2017 tumor-node-metastasis (TNM) classification [35] and nuclear grading [36]. A board certified, well-experienced pathologist evaluated histological diagnosis and the suitability of the tissue sections. Tumor central and margin areas were selected, four µm thick slices were created and consecutive slides were used to compare the same field of view in each case.

### 2.3. Multiplex Immunofluorescence (IF) Staining

We carried out a multiplex immunolabeling for examination of CXCR3, CD4, CD8, CD68 and cytokeratin with staining of thin FFPE sliced tumor sections. Multiplex immunohistochemistry was performed using Opal 6-plex Detection Kit (cat: NEL821001KT, Akoya Biosciences, Menlo Park, CA, USA). A multiplex panel of immune markers was developed with antibodies against following: CXCR3 (clone EPR25373-32, cat: ab288437, dilution 1:200, Abcam, Cambridge, MA, USA), CD4 (clone EP204, cat: 104R-26, dilution 1:50, Cell Marque), CD8 (clone C8/144B, cat: M710301-2, dilution 1:200, Dako/Agilent, Santa Clara, CA, USA), CD68 (clone PG-M1, cat: M087601-2, dilution 1:250, Dako/Agilent), FoxP3 (clone 236A/E7, cat: ab20034, dilution 1:150, Abcam), cytokeratin (clone AE1/AE3, cat: MA5-13156, dilution 1:500, Thermo-Fisher, Waltham, US). The staining procedure was performed using an automated staining system (BOND-RX; Leica Biosystems, Vienna, Austria) and included several cycles. All primary antibodies were sequentially applied (incubation time 30 min, RT), follow by the secondary polymerized reporter enzyme staining solution (incubation time 10 min, RT) and paired with respective Opal fluorophores (Table 1).

To visualize cell nuclei, the tissue was stained with 4‘,6-diamidino-2-phenylindole (spectral DAPI, Akoya Biosciences, Marlborough, US). Slides were scanned at 20× magnification using Mantra 2 Quantitative Pathology Workstation (Akoya Biosciences) and representative images from each tissue were acquired with the Mantra Snap software Version 1.0.4. Image spectral deconvolution, multispectral image analysis and cell phenotyping was carried out using the InForm Tissue Analysis Software Version 2.4.10 (Akoya Biosciences). Each of the images was reviewed and manually curated to assure no artefacts were included. In short, DAPI staining was used to detect nuclei and segment cells. The perinuclear area, defined as 4-pixel area around the nuclei, was considered as the cell cytoplasm area. Hereafter, the total cell area was evaluated for nucleic, cytoplasmic and membrane marker expression, respectively. The inForm build-in algorithm for cell phenotyping was used to manually define cells positive to each of the markers. The intensity of the marker expression in selected cells was used to set the thresholds for marker positivity and each cell was characterized and phenotyped by presence or absence of the marker. Immune cell infiltration was evaluated as the number of cells per analyzed tissue area.

**Figure 1:**
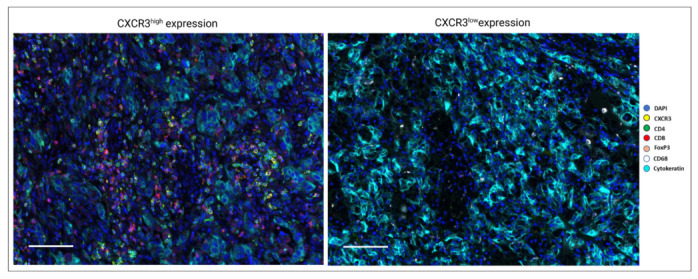
Multiplex immunofluorescence images presenting high and low CXCR3 infiltrated clear-cell renal cell carcinoma (CXCR3^high^ vs. CXCR3^low^). Scale bar = 100 μm.

We distinguished tumors with high and low CXCR3 expression, namely CXCR3high and CXCR3low, according to the median value. Representative stains for defined high and low CXCR3 expression in clear-cell RCC is shown in Figure 1.

**Figure 2:**
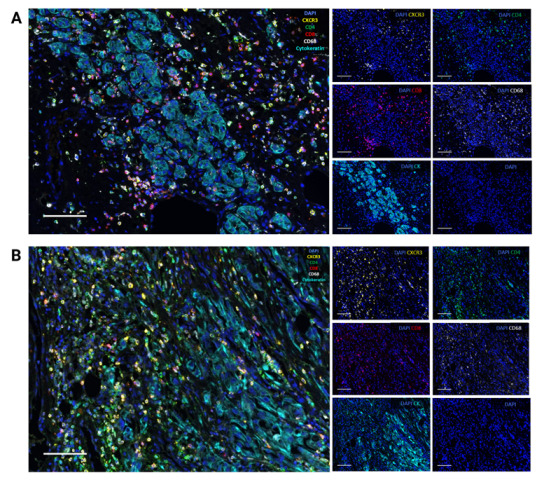
Representative multiplex immunofluorescence image of highly infiltrated (**A**) clear-cell renal cell and (**B**) papillary renal cell carcinoma displaying cells expressing chemokine receptor CXCR3, CD4, CD8, CD68, and pan-cytokeratin together with DAPI. Scale bar = 100 μm.

Spectral signatures were obtained and captured, creating figures from fluorescent images in one collective image and the same boxed region for each single fluorescent signal representing CXCR3, CD4, CD8, CD68, cytokeratin and solely DAPI in ccRCC, Figure 2A, and pRCC, Figure 2B.

### 2.4. Single-Cell RNA-Seq Analysis

The respective dataset consisting of samples obtained from 12 patients with RCC was downloaded as AnnData object (h5ad) from previously published studies of Li et al. [37] (Dataset [38]) and imported in Scanpy Version 1.9.1. The dataset was controlled for the quality with scanpy by thresholding the number of detected genes (200), counts (2000) and the fraction of mitochondrial reads (<30%). 

Cell transcriptomes were embedded into a batch-corrected low-dimensional latent space using scVI [39,40] treating each sample as a separate batch. The scVI model was trained on the 2000 most ‘highly variable genes’ as determined with scanpy’s “pp.highly_variable_genes‘ with parameters ‘flavor=”seurat‘‚ and batch_key=‘orig.ident‘. A neighborhood graph and UMAP embedding was computed based on the scVI latent space. All cell-type annotations were used from the original study [37]. Annotated cell types were confirmed by a set of cell type–specific markers such as, CD3E, CD68, CD8A, CD4, CD79A, KIT, CDH5, ACTA2, EPCAM. For more detailed analysis ‘tumour’ and ‘normal-kidney’ samples were extracted from the dataset and marged into a sparate AnnData object. 

Data analysis and graphical visualization was performed with scanpy v.1.9.1, anndata v.0.8.0, umap v.0.5.3, numpy v.1.21.5, scipy v.1.7.3, pandas v.1.4.2, scikit-learn v.1.02.2, statsmodels v.0.13.2, pynndescent v.0.5.7, and python-igraph v.0.10.2. 

### 2.5. Statistical Analysis

Result values are presented as means with standard deviation (±SD) and as frequencies with percentages for continuous variables. Variables between groups were compared using the Chi-square test and paired *t*-test. Correlation analysis was performed by Spearman’s ρ correlation coefficient (*r*_s_). The median value of CXCR3 and immune cell subset expression was used as cutoff value to divide patients into two low versus high expression groups for *t*-testing and Kaplan-Meier survival analysis. Survival curves representing time to RFS and OS were estimated using Kaplan-Meier curves with log-rank testing. All statistical comparisons were two-tailed with *p*-values < 0.05 considered as statistical significance. All analysis were performed using GraphPad (Version 9.0.0 for Windows, GraphPad Software, San Diego, California, USA). Graphical figures were created with GraphPad (Version 9.5.0 for Windows, GraphPad Software, San Diego, California, USA) and Biorender (Toronto, ON, Canada).

## 3. Results

### 3.1. Patient and Tumor Characteristics

Forty-two patients were included of which tumor samples were analyzed, mean (±SD) age was 62.1 (±12.3) years, 73.8% (*n* = 31) were of male sex and mean FU time was 96.4 (±57) months. 19% (*n* = 8) of all included patients were active cigarette smokers, showing no difference between non-smokers and smokers regarding further tumor recurrence. Furthermore, there was no significant difference in RCC recurrence regarding the presence of chronic kidney disease > grade 3a, type II insulin-dependent diabetes mellitus or the intake of alcohol on a regular basis, defined as >3 times per month. 59.5% (*n* = 25) were classified as ccRCC and 40.5% (*n* = 17) as non-clear cell renal carcinoma (nccRCC). There was no difference in rate of recurrence between the histological subtypes. Of all patients, 27 (64.3%) and 15 (35.7%) presented with the primary tumor staged pT1/T2 and pT3/T4, respectively. Twenty-nine (69%) patients were classified as grade 1 or 2 (G1/G2), 13 patients (31%) had tumor grade 3 or 4 (G3/4).

20 (90.9%) patients with no recurrence were tumor stage pT1/2, with the remaining 9.1% (*n* = 2) being pT3/4, showing statistical significance (*p* = 0.0003). There is also a significant difference in the onset of tumor recurrence according to tumor grade with 11 individuals being affected in tumors G3/4 and only nine with tumor grade G1/2. Mean CXCR3 expression was significantly higher in patients with tumor recurrence compared to recurrence-free individuals (113.3 vs. 36.4, *p* = 0.0251). There were no differences in expression seen in expression of CD4, CD8, CD68 and FoXP3 between the recurrence and non-recurrence group. Table 2 describes descriptive values in the ratio of recurrence to non-recurrence. 

### 3.2. CXCR3 Expression and Histopathological Characteristics

**Figure 3:**
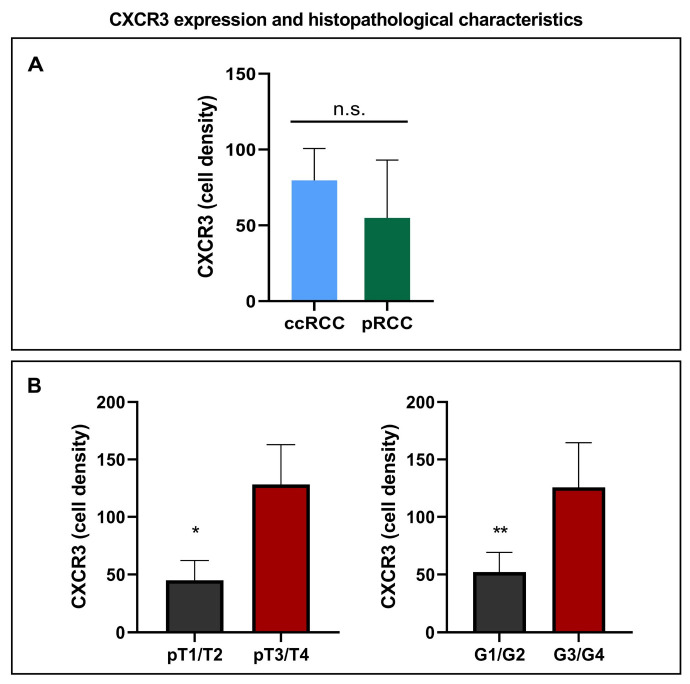
(**A**) shows no difference in CXCR3 expression when comparing the histopathological groups of ccRCC and pRCC. A significant higher expression of CXCR3 could be demonstrated in tumors staged pT3 or 4 and a nearly significant higher expression of CXCR3 in tumor grade 3 or 4 (**B**). *p* values by log-rank test; * *p* = 0.0044, ** *p* = 0.0518. ccRCC = clear-cell renal cell carcinoma, pRCC = papillary renal cell carcinoma.

Next, we explored the histological subtype-specific differences in CXCR3 expression. There was no difference in cell density of CXCR3 between the histological subtypes of clear-cell RCC and papillary RCC, Figure 3A. Statistical significance was seen in higher cell density of CXCR3 and tumors staged pT3/4 (*p* = 0.0044) and near significance was observed in CXCR3 expression comparison with tumor grade G1/2 to G3/G4 (*p* = 0.0518, Figure 3B). 

### 3.3. CXCR3 Is Mainly Expressed on CD8+ and CD4+ T Cells According to Single-Cell RNA-Seq and Multiplex Immunofluorescence

**Figure 4:**
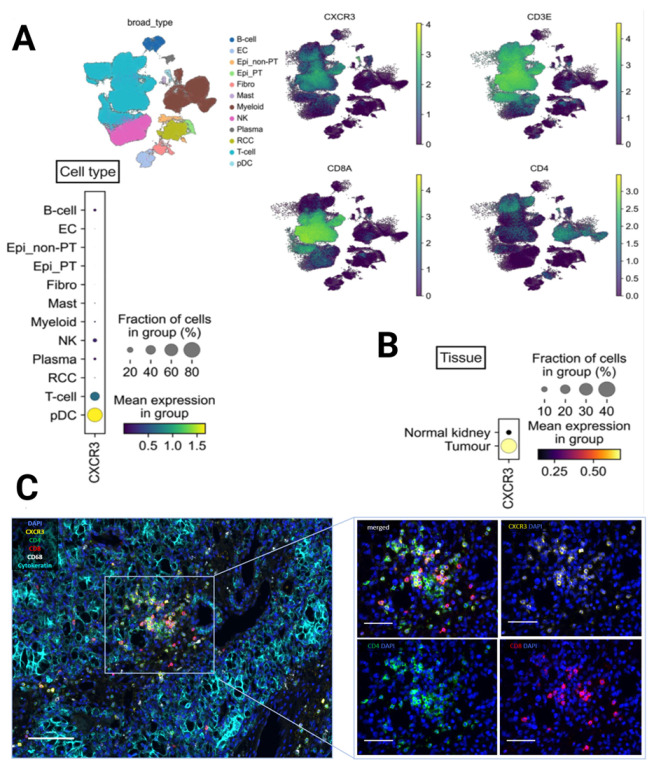
Single-cell analysis reveals CXCR3 mRNA expression on CD8 and CD4 T cells, and dendritic cells in a publicly available RCC dataset. (**A**) Integrated UMAP for CXCR3, CD3E, CD4 and CD8A, and the respective annotated cell types from dissociated primary tumors obtained from patients with RCC (*n* = 12). Strong CXCR3 gene expression was found in the T cells and dendritic cells. (**B**) T cells expressing CXCR3 were mainly localized in the tumor compared to the normal renal tissue. (**C**) Multiplex immunofluorescence image of renal cell carcinoma (RCC) showing co-expression of chemokine receptor CXCR3 with CD8 and CD4 T cells. Scale bar = 100 μm and magnification of regions representing single infiltration. Scale bar = 50 μm.

Single-cell RNA-seq analysis of annotated cell clusters from 12 patients with RCC revealed CXCR3 expression mainly in T cells (around 30–40%), and dendritic cells. Most of dendritic cells in the tissue express CXCR3 as well, but the cell population is very small, in comparison to T cell population (Figure 4A). In-depth analysis of CXCR3 expression showed that T cells expressing CXCR3 are predominantly located in the tumor region in comparison to normal kidney (Figure 4B). Multiplex IF experiments of in-house RCC samples (*n* = 42) also confirmed that CXCR3 is predominantly expressed on CD8+ and CD4+ T cells (Figure 4C). 

**Figure 5:**
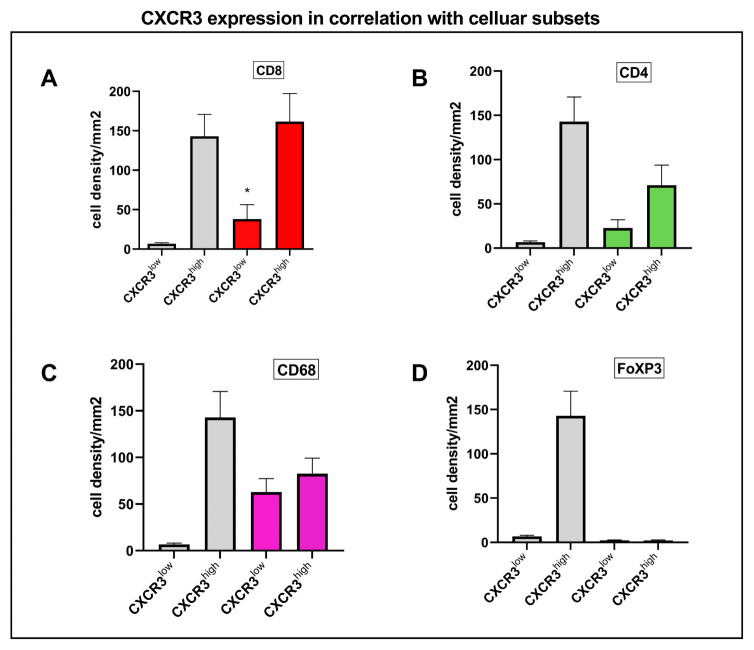
The overall CXCR3 expression of the sample’s cohort, divided into CXCR3^low^ and CXCR3^high^, is shown as a reference value in all subgraphs (**A**–**D**), grey. The colored columns represent the expression of CXCR3 in different cellular subtypes, showing statistically significant higher CXCR3 expression in CD8 T-cells (**A**) (* *p* < 0.0001). CD4 (**B**) and CD68 (**C**) were higher expressed, yet without significance and FoPX3 (**D**) showed no expression.

We further investigated whether either low or high CXCR3 expression was associated with cell density of CD8, CD4, CD68 and FoXP3 expression in tumor infiltrating lymphocytes (TILs) and found a significant correlation between CD8+ T cell infiltration and CXCR3high tumors (*p* < 0.0001) compared to CXCR3low tumors, Figure 5A. CD4 (Figure 5B) and CD68 (Figure 5C) were found to be higher expressed in case of an underlying CXCR3high tumor, yet without statistical significance. 

### 3.4. Correlation Analysis of CXCR3 and Immune Cell Subsets

A statistically significant correlation was observed between the expression of CXCR3 and CD8^+^ (*r_s_* = 0.515, *p* < 0.001) T cells, and CXCR3 and CD4^+^ T cells (*r_s_* = 0.432, *p* = 0.0043, yet it is to bear in mind that the *r_s_* correlation coefficient remains to be not very high, indicating a only moderate strength of correlation. 

**Figure 6:**
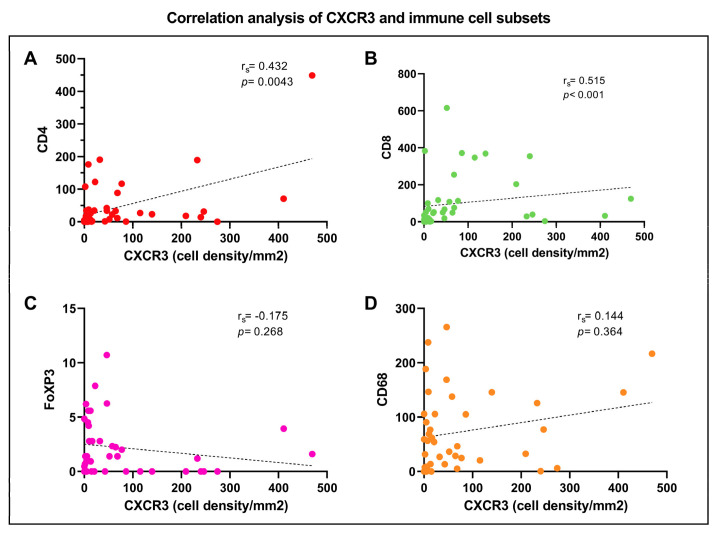
Spearman’s correlation coefficient and *p*-values showing a significant correlation between CXCR3 expression and CD8 (**A**) and CD4 (**B**), a slight negative correlation between CXCR3 and FoXP3 (**C**) and no correlation evaluating CD68 and CXCR3 (**D**).

There was a negative correlation between expression of CXCR3 and FoXP3 and no correlation between CXCR3 and CD68, Figure 6A–D, respectively. 

**Figure 7:**
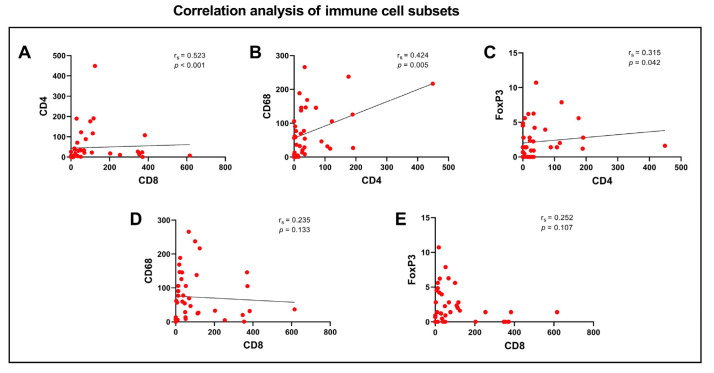
Spearman’s correlation coefficient and *p*-values showing a significant correlation between the infiltration density of CD8 and CD4 (**A**), CD4 and CD68 (**B**), CD4 and FoXP3 (**C**), but no coherence between CD8 and CD68 (**D**) or FoXP3 (**E**).

Furthermore, significant correlations were identified between presence of CD8 and CD4 (rs = 0.523, *p* < 0.001), CD4 and CD68 (rs = 0.424, *p* = 0.005) and CD4 and FoxP3 (rs = 0.315, *p* = 0.042), Figure 7A–C, respectively. 

### 3.5. Influence of CXCR3 Expression on Survival 

**Figure 8:**
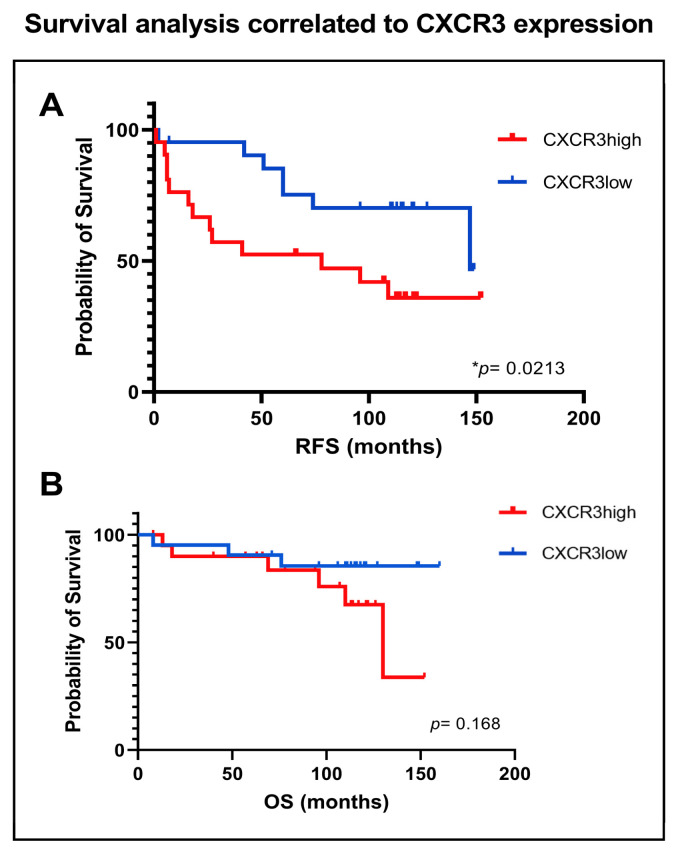
Association between low or high CXCR3 expression and survival. Kaplan-Meier survival curves comparing (**A**) recurrence-free survival (RFS), and (**B**) overall survival according to high or low CXCR3 expression (CXCR3^low^ vs. CXCR3^high^). *p* values by log-rank test. * *p* value < 0.05.

Survival analysis revealed that patients in the CXCR3^high^ tumor group experience a significant shorter time to RFS compared to the CXCR3^low^ group (median 78 vs. 147 months, *p* = 0.0213), Figure 8A. OS did not significantly differ between the two groups (*p* = 0.168), Figure 8B. 

**Figure 9:**
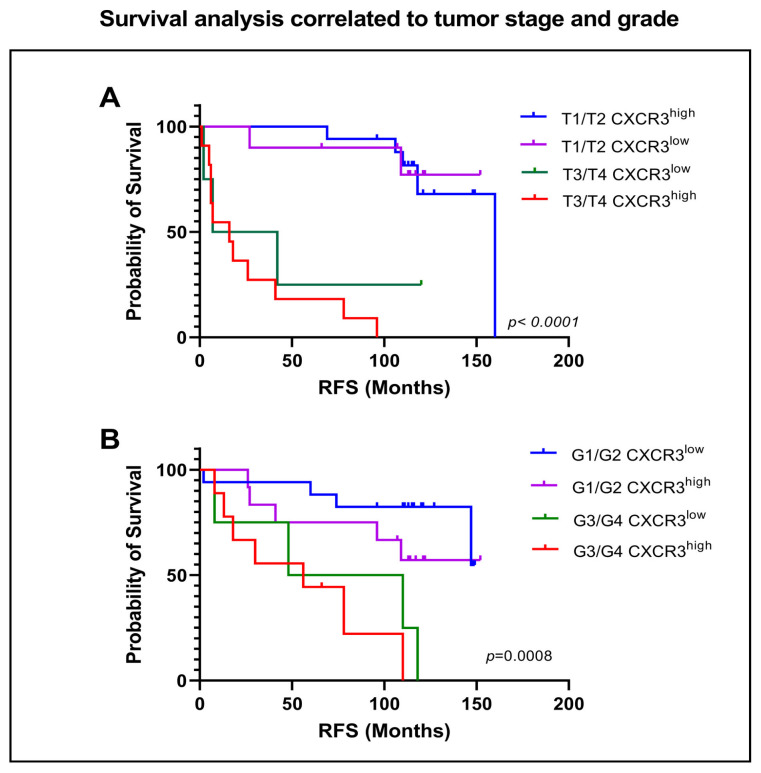
Association between tumor grade and tumor stage set in correlation with low or high CXCR3 expression and survival. Kaplan-Meier survival curves comparing (**A**) recurrence-free survival (RFS) in terms and correlated to tumor stage and (**B**) tumor grade. *p* values by log-rank test.

We then compared the groups of each pT1/2 and pT3/4 staged tumors with each CXCR3^low^ and CXCR3^high^ expression. RFS was significantly shorter in the pT3/4 CXCR3^high^ RCC group compared to the pT1/2 CXCR3^high^ group with 16 vs. NR months (*p* < 0.0001). Noticeable, within the same group of RCCs staged pT3/4, a further timely difference in RFS between CXCR3^high^ and CXCR3^low^ expression has become apparent (median 16 vs. 24.5 months), Figure 9A. The same trend was observed when focusing on tumor grade, G1/2 tumors have still no reached RFS, irrespective of their CXCR3 status. CXCR3^high^ tumors graded G3/4 had a RFS of 56 months, showing statistical significance in relation to grade G1/2 CXCR3^high^ (*p* = 0.0008). Comparison of RCCs graded G3/4 with either CXCR3^high^ or CXCR3^low^ expression showed a not to been overseen difference in time to RFS (median 56 vs. 79 months), Figure 9B.

Focusing on immune cell subsets, patients with CD8^high^ expression exhibited no significant difference in RFS compared to CD8^low^ expression. The same could be observed for comparison of CD4^high^/CD4^low^, CD68^high^/CD68^low^ and FoxP3^high/^FoxP3^low^, as seen in Figure S1. 

## 4. Discussion

Biomarkers in predicting early recurrence after primary surgical resection of non-metastatic RCC are still lacking. Studies up to date are all based on the TNM staging system, histological subtype and histological grade, known as prognostic histopathological parameters in RCC. To the best of our knowledge, for the first time, this study assessed the expression and correlation of CXCR3 and immune cell subsets such as CD8, CD4, FoxP3 and CD68 expression in samples of surgically treated non-metastatic RCC. We investigated their potential prognostic value separately and in correlation to each other to define a potential biomarker to stratify patients at high risk, profiting the most from adjuvant therapy. 

Our multiplex IF and single-cell analysis showed, that CD8+ and CD4+ T cell-infiltrating RCC cells have a high rate of CXCR3 expression. Moreover, our results also confirm that, in addition to tumor stage and grade, high CXCR3 expression correlates with a significant shorter time to recurrence of disease. The role of CXCR3 in tumor development remains complex, possibly depending on the isoform, cell type and microenvironment in which the receptor is expressed in. CXCR3 expression in our cohort was independent of histological subtype. However, there was a significant higher expression of CXCR3 with higher tumor stage (pT3/T4) and higher tumor grade (G3/G4), being in line with literature to date reporting that overexpression of IFN-inducible CXCR3 ligands predicted poorer oncological outcomes in the past [28]. CD8+ T cell infiltration in RCC is also related to poorer prognosis [41,42] which was found to be significantly dependent in co-expression with CXCR3 as shown by correlation analysis. Our correlation analysis additionally illustrates a significant high correlating co-expression of CD8 in CXCR3high tumor cells, underlining the interaction of the two cellular regulators with both referring to a worse disease outcome. Data from Bangs et al. have moreover shown that CXCR3 is likely to play a role in the proliferation of T cell factor 1 precursor [17], also aligning the described requirement for CXCR3 to achieve effective checkpoint blockade treatment [43]. Expression of CXCR3 ligands interact with vascular cell adhesion molecules to mediate CD8^+^ T cells to malignant tissue [44]. These findings were underlined by Gunderson et al. demonstrated the correlation of CXCR3 and CD8^+^ T cells by describing a reverse mechanism of immunosuppression through inhibition of CXCR3 in CD8+ T cells, thereby limiting their trafficking into tumors [45] underlining the importance of the CXCR3-CD8^+^ T interaction. Further analysis also evinced that CD8 and CD4 positivity correlate in a significant degree. Additionally, CD4 has shown to have a high correlation to CD68 and FoxP3, which could mean that expression of all these regulatory cells is in the context of a worse oncological outcome, which is in line with current literature [46,47]. Positivity in CD8 on T cells is also known to predicted prognosis and effect of antiangiogenic treatments [42] and enhancement of CD8 cell response by PD-1 blockade has also shown to be critical for the efficacy of immunotherapy [48]. 

On base of the Keynote 564 trial, pembrolizumab was approved as adjuvant therapy for patients after surgery of localized kidney cancer with a high risk of recurrence by the FDA and EMA [8]. The study reveals a very wide range of included patients all defined as ‘high risk for recurrence’, as patients with a defined intermediate-high and high risk of tumor recurrence were included. A closer look at the inclusion criteria illustrates the following risk definitions with known disease-free survival according to the UCLA Integrated Staging System (UISS) [43]. Intermediate-high risk was defined as tumor stage pT2 with nuclear grade 4 or sarcomatoid differentiation (5-year DFS 80%) or tumor stage 3 with any tumor grading and without lymph node invasion or distant metastasis (5-year DFS 55–80%). High risk patients included tumor stage pT4 of any grade without lymph node invasion or distant metastasis (5-year DFS 55%), any tumor stage with regional lymph node invasion (5-year DFS 32%) as well as metastatic patients with no evidence of disease after resection of oligometastatic sites < one year from nephrectomy (3-year DFS 20% [49]). In summary, one could hypothesize that the inhomogeneous cohort of patients in the study does not provide a reliable indication of which patient benefits the most from adjuvant therapy. Currently, phase III randomized controlled trials are ongoing, evaluating the efficacy of immunotherapy in the adjuvant setting of resected RCC (RAMPART (NCT03288532), PROSPER (NCT03055013) and NCT05239728). These studies also rely on risk stratification on base of the TNM staged without additionally usage of a potential path-leading biomarker. A far-reaching goal with regards to these studies would be to declare a clinical applicable biomarker for high risk of recurrence, according to which patients could be specifically recruited. 

Chow et al. described that CXCR3-deficient mice responded poorly to anti-PD-1 treatment and that CXCR3 expression was critical for a functional CD8 T-cell response in tumors treated with checkpoint inhibition [50]. CXCR3 ligands additionally were an indicator of clinical response to anti PD-1 in patient plasma, indicating that the CXCR3 chemokine is a biomarker for efficacy to PD-1 blockade [50]. Survival analysis of our data reveal that patients with CXCR3high tumors had a significant shorter time to recurrence compared to CXCR3low tumors with a median time of 78 vs. 147 months, respectively. Survival analysis of other immune cell subsets, such as CD8, CD4, CD68 and FoxP3 expression remained negative regarding RFS. 

To determine dependency of survival on tumor stage and grade combined with expression of CXCR3, further analysis was performed. As expected, patients with tumor stage pT3/4 or grade G3/4 showed significantly poorer RFS compared to lower stages. We subsequently put each low or high expression of CXCR3 into further correlation and could observe a considerable difference in time to recurrence when comparing pT3/4 and grade 3/4 with CXCR3low vs. CXCR3high (24.5 vs. 16 months and 79 vs. 56 months, respectively). The difference in the subpopulation group of pT1/2 CXCR3low with 160 months to recurrence versus the group staged T3/T4 with CXCR3high with 16 months describes one yet impressive effect of the influence of CXCR3 expression on the RFS. 

Chuah et al. identified circulating CXCR3 expressing CD8+ T cells as biomarkers for response to anti-PD-1 in patients with hepatocellular cancer, assigning CXCR3 not only to a role as prognostic marker of recurrence but also as a marker of response to anti-PD1 therapy [51]. Further, CXCR3 ligands are also seen as positive indicators of responsiveness to anti-PD-1 therapy [50]. CD8 T cell infiltration has additionally shown to be indicative to a benefit from combined anti–PD-1 and anti–CTLA-4 in patients with solid tumors [52] hypothesizing that high CXCR3 with highly co-expressed CD8 also could be beneficial when receiving immunotherapy. Similar results were described by Feng et al. in a study with metastatic bladder cancer, where activation of the CXCR3 pathway is proposed as a novel predictive biomarker for the efficacy of immunotherapy [53]. 

## 5. Conclusions

In conclusion, CXCR3 was mainly expressed on intratumoral CD8+ and CD4+ T cells. We have shown that high density and expression of CXCR3 is associated with advanced tumor stage and poorer RFS in surgically treated RCC. Our analysis might represent an additional prognostic signature that could constitute a novel indicator beyond TNM staging and histological grade. CXCR3 might therefore be a useful prognostic as well as predictive biomarker in selecting patients at high recurrence risk for adjuvant immunotherapy.

## Figures and Tables

**Table 1:** Table of Complete, Transparent, Accurate and Timely account (CTAT). List of applied antibodies with respective used reagents and software.

Antibodies				
Name	Citation	Supplier	Cat No.	Clone No.
CXCR3	IF	Abcam	ab288437	EPR25373-32
CD4	IF	Cell Marque	104R-26	EP204
CD8	IF	Dako	M710301-2	C8\144B
FoxP3	IF	Abcam	ab20034	236A/E7
CD68	IF	Dako	M087601-2	PG-M1
Cytokeratin	IF	Thermo Fisher	MA5-13156	AE1/AE3
**Software**				
**Name**	**Manufacturer**			**Version**
Mantra Snap	Akoya Biosciences			1.0.4
inForm Analysis	Akoya Biosciences			2.4.10
**Reagents & Materials**				
**Name**	**Supplier**			**Cat no.**
Opal 6-plex Detection Kit	Akoya Biosciences			NEL821001KT
BOND Polymer Refine Detection	Leica Biosystems			DS9800
BOND Epitope Retrival 1	Leica Biosystems			AR9961
BOND Epitope Retrival 2	Leica Biosystems			AR9640
BOND Dewax Solution	Leica Biosystems			AR9222
BOND Wash Solution 10×	Leica Biosystems			AR9590
Spectral DAPI	Akoya Biosciences			FP1490
Prolong Diamond Antifade	Thermo Fisher			P36961
BOND Research Detection System	Leica Biosystems			DS9455
BOND Titration Kit	Leica Biosystems			OPT9049
Opal 6-plex Detection Kit	Akoya Biosciences			NEL821001KT
BOND Polymer Refine Detection	Leica Biosystems			DS9800
BOND Epitope Retrival 1	Leica Biosystems			AR9961
BOND Epitope Retrival 2	Leica Biosystems			AR9640

**Table 2:** Descriptive patient characteristics at diagnosis and tumor characteristics. SD = standard deviation, BMI = Body Mass Index, CKD = chronic kidney disease, IDDM = insulin-dependent diabetes mellitus. # *p* values from Fisher’s exact test for categorical variables, and independent *t*-tests for quantitative variable.

Factor	Total (*n* = 42)	Recurrence (*n* = 20)	No Recurrence (*n* = 22)	*p* Value ^#^
Age (years), mean (SD)	62.1 (12.3)	61 (10.2)	63.1 (14.1)	0.588
Male sex, *n* (%)	31 (73.8%)	17 (85%)	15 (68.2%)	0.284
BMI, mean (SD)	26.0 (4.6)	25.7 (4.7)	26.4 (4.5)	0.472
Cigarette Smoking, *n* (%)	8 (19%)	3 (15%)	5 (22.8%)	0.700
CKD > grade 3a, *n* (%)	6 (14.2%)	1 (5%)	5 (22.8%)	0.187
IDDM type II, *n* (%)	8 (19%)	5 (25%)	3 (13.6%)	0.445
Alcohol intake > 3×/month	23 (54.7%)	9 (45%)	12 (54.5%)	0.232
pT, *n* (%)				0.0003
pT1/T2	27 (64.3%)	7 (35%)	20 (90.9%)	
pT3/T4	15 (35.7%)	13 (65%)	2 (9.1%)	
Tumor grade, *n* (%)				0.002 **
G1/G2	29 (69%)	9 (45%)	20 (90.9%)	
G3/G4	13 (31%)	11 (55%)	2 (9.1%)	
Histology, *n* (%)				1.000
ccRCC	25 (59.5%)	12 (60%)	13 (59.1%)	
nccRCC	17 (40.5%)	8 (40%)	9 (40.9%)	
Sarcomatoid differentiation, *n* (%)	2 (4.8%)	2 (10%)	0 (0%)	0.527
CXCR3, mean (SD)	74.8 (112.7)	113.3 (141.1)	36.4 (55)	0.0251 *
CD4, mean (SD)	46.9 (82.4)	38.8 (99)	54.3 (65.2)	0.548
CD8, mean (SD)	99.8 (142.3)	120.3 (172.6)	81.3 (108.7)	0.381
CD68, mean (SD)	72.8 (70.9)	72.1 (80.3)	73.4 (63.1)	0.957
FoXP3, mean (SD)	2.2 (2.6)	1.7 (2.8)	2.6 (2.3)	0.263
Follow-up (months), mean (SD)	96.4 (57)	76.2 (44.8)	114.5 (22)	0.001 **

* *p* < 0.05, ** *p* < 0.01.

## Data Availability

The data that support the findings of this study are available from the database of our institution, responsibility for the integrity of the data and the accuracy of the data is taken by the first and corresponding author.

## Supplementary Materials

The following supporting information can be downloaded at: https://www.mdpi.com/article/10.3390/cancers15041001/s1, Figure S1: Kaplan-Meier survival curves showing (A) recurrence-free survival (RFS), and (B) overall survival according to high or low CXCR3 expression (CXCR3^low^ vs. CXCR3^high^). *p* values by log-rank test.
